# Identification of a novel zinc-binding protein, C1orf123, as an interactor with a heavy metal-associated domain

**DOI:** 10.1371/journal.pone.0204355

**Published:** 2018-09-27

**Authors:** Yoshiaki Furukawa, Carolyn Lim, Takehiko Tosha, Koki Yoshida, Tomoaki Hagai, Shuji Akiyama, Shoji Watanabe, Kenta Nakagome, Yoshitsugu Shiro

**Affiliations:** 1 Department of Chemistry, Keio University, Yokohama, Kanagawa, Japan; 2 RIKEN SPring-8 Center, Sayo, Hyogo, Japan; 3 Research Center of Integrative Molecular Systems (CIMoS), Institute for Molecular Science, NINS, Okazaki, Aichi, Japan; 4 Department of Functional Molecular Science, SOKENDAI (The Graduate University for Advanced Studies), Okazaki, Aichi, Japan; 5 Graduate School of Brain Science, Doshisha University, Kyotanabe, Kyoto, Japan; 6 Graduate School of Life Science, University of Hyogo, Ako, Hyogo, Japan; University of Saskatchewan, CANADA

## Abstract

Heavy metal-associated (HMA) domains bind metal ions at its Cys-x-x-Cys (CxxC) motif and constitute an intracellular network for trafficking of metal ions for utilization and detoxification. We thus expect that novel metalloproteins can be identified by screening proteins interacting with a HMA domain. In this study, we performed yeast two-hybrid screening of the human proteome and found an uncharacterized protein encoded as open reading frame 123 in chromosome 1 (C1orf123) that can interact specifically with the HMA domain of a copper chaperone for superoxide dismutase (CCS^dI^). Our X-ray structural analysis of C1orf123 further revealed that it binds a Zn^2+^ ion in a tetrahedral coordination with four thiolate groups from two conserved CxxC motifs. For the interaction between C1orf123 and CCS^dI^, the CxxC motifs in both C1orf123 and CCS^dI^ were required, implying metal-mediated interaction through the CxxC motifs. Notably, C1orf123 did not interact with several other HMA domains containing CxxC motifs, supporting high specificity in the interaction between C1orf123 and CCS^dI^. Based upon these results, we further discuss functional and structural significance of the interaction between C1orf123 and CCS.

## Introduction

Heavy metal ions have essential roles in our lives, for example, by functioning as redox centers in protein electron transfers and active centers in enzymatic reactions. As represented by metal-related diseases, however, heavy metal ions can also exhibit quite high toxicity when mishandled in our bodies [1]. Cells are, therefore, equipped with a network of metal-binding proteins to transport, utilize, and detoxify metal ions [2].

A heavy metal-associated (HMA) domain is a simple protein architecture with ~70 amino acids that can bind heavy metal ions (copper and zinc ions in particular) [3]. The HMA domain has a βαββαβ folding pattern with a conserved Cys-x-x-Cys (CxxC) motif, in which the thiolate groups of the Cys residues function as ligands for binding metal ions [3]. A comparative analysis on the available genomes has identified HMA domains in metal-related proteins from bacteria to mammals [4], and most of the proteins containing a HMA domain(s) play central roles in transporting and intracellular trafficking of copper/zinc ions [3, 5]. For example, in budding yeast, Atx1, which is a copper chaperone composed of a single HMA domain, receives a cuprous ion from a copper importer, Ctr1, binds it through the CxxC motif [6, 7], and then delivers it to one of the HMA domains in the copper-transporting ATPase, Ccc2 [8]. The specific interaction of Atx1 with Ccc2 mediated by a copper ion has been extensively studied at atomic resolution, showing that the cuprous ion is equilibrated between Atx1 and Ccc2 by exchanging thiolate ligands from their respective CxxC motifs [8–10]. Mutations in human homologues of Ccc2, ATP7b and ATP7a, disturb the trafficking of copper ions, causing Wilson and Menkes diseases due to abnormal accumulation and deficiency of copper ions, respectively [11]. Therefore, protein-protein interactions involving HMA domains are critically important in handling toxic heavy metal ions safely in our bodies.

Given that proteins with HMA domains behave as a hub for intracellular trafficking of metal ions by delivering and receiving them, we expect that screening of proteins interacting with HMA domains would identify intracellular metalloproteins. For this purpose, we used in this study a copper chaperone for superoxide dismutase, CCS, which is a protein responsible for supplying a copper ion and also introducing an intramolecular disulfide bond to Cu/Zn-superoxide dismutase (SOD1) [12–14]. CCS is composed of three distinct domains, and the N-terminal domain 1 (CCS^dI^) is a HMA domain [15]. CCS^dI^ can tightly bind a cuprous ion at its CxxC motif [16, 17], but the CxxC motif in CCS^dI^ is not required for the copper transfer from CCS to SOD1, which was demonstrated by the result that substitution of Ala for Cys residues in the CxxC motif had no effect on the copper transfer to SOD1 [18, 19]. Indeed, supplying a copper ion to SOD1 is known to be performed by a C-terminal domain 3 of CCS (CCS^dIII^) [20, 21]. Also, CCS^dI^ appears not to be involved in the recognition of SOD1, for which a central domain 2 of CCS (CCS^dII^) is responsible [20, 21]. Therefore, exact roles of CCS^dI^ in the function of CCS still remain obscure, and to date, no known interactors of CCS^dI^ have been identified.

In order to identify intracellular metalloproteins, we performed yeast two-hybrid screening of the human proteome using CCS^dI^ as bait and found that an uncharacterized protein encoded as open reading frame 123 in chromosome 1 (C1orf123) interacted specifically with CCS^dI^. The crystal structure revealed that C1orf123 binds a Zn^2+^ ion in a tetrahedral coordination with four Cys residues from two CxxC motifs. Based upon the results, we will further discuss functional and structural significance of the interaction between C1orf123 and CCS.

## Materials and methods

### Yeast two-hybrid screening of protein-protein interactions

Yeast two-hybrid screening was performed by using Matchmaker^TM^ Gold Yeast Two-Hybrid System (Clontech). cDNA of CCS^dI^ was cloned into a plasmid vector, pGBK-T7, with which a Y2H-Gold yeast strain was transformed. The Y2H-Gold yeast strain was then mated with a Y187 yeast strain containing a normalized library of human cDNAs in pGAD-T7 plasmid vectors (Clontech; Mate & Plate^TM^ Library–Universal Human (Normalized)). Mated yeast cells were screened on plates containing double dropout medium (DDO: Synthetic defined media lacking Leu and Trp) with an antifungal, Aureobasidin A (AbA), and a metabolite, 5-bromo-4-chloro-3-indolyl-α-D-galactopyranoside (X-α-Gal). According to the manufacturer’s instruction, pGAD-T7 plasmids were isolated from each of the blue colonies grown on the plates, and the cDNAs cloned in the isolated pGAD-T7 were sequenced.

The yeast two-hybrid system was also used for the analysis of distinct protein-protein interactions. Briefly, a Y2H-Gold yeast strain was transformed with pGBK-T7 harboring cDNA of a protein with a heavy metal-associated domain such as Atx1, HAH1, Ccc2a, and CCS, while a Y187 yeast strain was transformed with pGAD-T7 harboring a cDNA of C1orf123. Those two yeast strains were mated and spotted on DDO plates with AbA and X-α-Gal, and protein-protein interactions were detected by the presence of blue colonies. Plates were incubated at 30 ^o^C for three days.

### Protein preparation

cDNA coding full-length C1orf123 was amplified with PCR from a library of human brain cDNAs and cloned into a pET-15b plasmid vector (Novagen). Mutations were introduced by inverse PCR using KOD-FX-Neo DNA polymerase (TOYOBO). All constructs used in this study were confirmed by DNA sequencing.

*E*. *coli* BL21(DE3) was transformed with the cloned plasmid vector, and the expression of C1orf123 with an N-terminal 6x His-tag was induced by culturing the *E*. *coli* cells with 0.1 mM isopropyl β-D-1-thiogalactopyranoside at 20 ^o^C for 20 hours. Cells were lysed in phosphate buffered saline (PBS) containing 2% Triton X-100 with 7.2 mg/L deoxyribonuclease I and 0.5 mM MgSO_4_ by freeze-thaw cycles and ultrasonication. The supernatant obtained by centrifugation of the lysate at 20,000 x *g* was loaded on a cOmplete^TM^ His-Tag Purification column (1 mL, Roche). After washing with a buffer containing 50 mM Tris and 100 mM NaCl at pH 7.0 (TN buffer), proteins with an N-terminal His tag were eluted from the column with TN buffer containing 100 mM imidazole. For removal of the N-terminal His tag, the eluted samples were concentrated in the presence of 5 mM dithiothreitol and incubated with HRV3C protease at 4 ^o^C for 16 hours. The sample was then re-loaded on a cOmplete^TM^ His-Tag Purification column (1 mL, Roche) to remove His tags and HRV3C. The flowthrough was collected, concentrated, and then purified with a gel-filtration column (COSMOSIL 5Diol-300-II, Nacalai Tesque) equilibrated with TN buffer. Successful purification of C1orf123 with removal of the N-terminal His tags was confirmed by SDS-PAGE, and the protein concentration was determined spectroscopically with the extinction coefficient at 280 nm (23,950 cm^-1^M^-1^).

### Molecular analysis of C1orf123

Secondary structures of C1orf123 were analyzed with Fourier transform infrared (FT-IR) spectroscopy. Buffer solution in the C1orf123 sample was first exchanged to TN buffer prepared using D_2_O with a centrifugal filtration device, and the sample was concentrated to 1 mM. The FT-IR spectra were then measured by using an IRAffinity-1S spectrophotometer (Shimadzu) attached with an attenuated total reflection module (DuraSamplIR II, nine reflections). IR peaks were assigned for secondary structures based on the work of Byler and Susi [22].

For the measurement of far-UV circular dichroism (CD) spectra using a CD spectrometer (J-720WI, Jasco), 20 μM C1orf123 was prepared in a buffer containing 10 mM sodium phosphate (Na-Pi) and 100 mM NaCl at pH 7.0.

The molecular size of C1orf123 in solution was examined by size-exclusion chromatography with on-line multi-angle light scattering (SEC-MALS). C1orf123 (2 g/L) in TN buffer was loaded on a gel filtration column (TSKgel G2000SW, TOSOH) fitted to an HPLC system (Shimadzu), and the absorbance change at 280 nm of the elution was monitored. The molecular size of the protein eluted from the column was determined by multi-angle light scattering using miniDAWN TREOS (WYATT Technology) connected on-line to the HPLC system.

Analysis of the zinc content in C1orf123 samples was performed by graphite furnace atomic absorption spectroscopy using an ICE 3000 spectrophotometer (Thermo Fisher scientific).

### Crystallization and X-ray structural analysis

C1orf123 was crystallized at 4 ^o^C by the sitting drop vapor diffusion method in drops containing 1 μL of 18 mg/mL C1orf123 in TN buffer mixed with an equal volume of a reservoir solution of 2.0 M ammonium sulfate, 100 mM sodium acetate trihydrate, pH 4.5. Cubic crystals with 100 μm x 100 μm x 100 μm in size were obtained within one day, which was reproduced in another independent experiment. The crystals were soaked into a cryoprotectant solution of 32% (w/v) xylitol, 1.6 M ammonium sulfate, 50 mM sodium acetate trihydrate, pH 4.5, and were flash-cooled in liquid nitrogen. We also examined the crystallization of C1orf123 at 20 ^o^C but could not obtain any crystals.

X-ray diffraction data were collected at 100 K at a wavelength of 1.0 Å on beamline BL26B2 at SPring-8 using a MX225HE CCD detector (Raynix). The data were processed using HKL2000 [23]. Statistics of the data collection are summarized in Table 1. Phases were calculated from zinc anomalous scattering by the SAD method (f’ = 0.24 and f” = 2.44) using PHENIX AutoSol [24]. Based upon the calculated electron density map, the initial model was built using ARP/wARP [25]. Further model building and refinement were performed with Refmac5 [26] and COOT [27]. The resulting final model, which consists of one polypeptide chain with a zinc atom and 175 water molecules, had an *R*_work_/*R*_free_ of 0.180/0.233. The refinement statistics are summarized in Table 1. The electrostatic potential of the surface of C1orf123 was calculated using APBS [28]. Coordinates and structure factor have been deposited under accession code 5ZLQ for C1orf123.

**Table 1:** Data collection and refinement statistics.

	C1orf123
**Data collection**	
Wavelength (Å)	1.0
Resolution (Å) ^a^	50.0–2.0 (2.03–2.00)
Space group	*I*23
Cell dimensions	
*a* = *b* = *c* (Å)	118.13
*α* = *β* = *γ* (°)	90.00
Observed reflections	828,155
Unique reflections	18,717
*R*_merge_ ^a^^,^ ^*b*^	0.067 (0.727)
*CC*_1/2_ ^c^	(0.975)
Completeness (%) ^a^	99.9 (100)
Redundancy ^a^	44.2 (42.1)
*I*/sigma(*I*) ^a^	69.3 (7.2)
**Refinement**	
*R*_work_ / *R*_free_ ^d^	0.180/0.233
No. atoms	
Protein	1,256
Ligand/ion	1
Water	175
Mean *B*-factors (Å^2^)	
Protein	26.7
Ligand	22.5
Water	33.5
R.m.s. deviation	
Bond length (Å)	0.020
Bond angles (°)	1.94
DPI (Å) ^e^	0.14
Ramachandran plot	
Favored region (%)	96
Outlier region (%)	0

^*a*^ Values in parenthesis are for the highest resolution shell.

^*b*^ *R*_merge_ = Σ_*hkl*_Σ_*i*_|*I*_*i*_(*hkl*) − <*I*(*hkl*)>| / Σ_*hkl*_Σ_*i*_*I*_*i*_(*hkl*), where <*I*(*hkl*)> is the average intensity of *i* observations.

^*c*^ Pearson’s correlation coefficient between average intensities of random half data sets for each unique reflection.

^*d*^ *R*_work_ = Σ_*hkl*_|*F*_obs_(*hkl*) − *F*_calc_(*hkl*)|/Σ_*hkl*_*F*_obs_(*hkl*), where *F*_obs_ and *F*_calc_ are the observed and calculated structure factors, respectively. *R*_free_ was calculated with 5% of the reflections.

^*e*^ Diffraction-data precision indicator (DPI) was calculated using Sfcheck.

## Results and discussion

### Yeast two-hybrid screening of human proteins interacting with CCS^dI^

To search for proteins that can interact with human CCS^dI^, yeast two-hybrid screening experiments were performed using a human cDNA library. In this screening system (Matchmaker^TM^ Gold Yeast Two-Hybrid System, Clontech), CCS^dI^ is the bait that is expressed as a fusion protein with the DNA binding domain of GAL4 transcription factor (GAL4 DNA-BD). Prey proteins are fused with the activation domain of GAL4 (GAL4 AD); therefore, interaction of prey with CCS^dI^ enables to trigger the transcription of reporter genes regulated by GAL4 promotor. The selection markers coded by those reporter genes include an Aureobasidin A (AbA) antibiotic resistance marker and a blue/white selection marker (α-galactosidase). Yeast cells with cDNA clones positive for the interaction with CCS^dI^ will thus be grown as blue colonies in the presence of AbA.

Expression of CCS^dI^ fused with GAL4 DNA-BD in yeast cells was confirmed by Western blotting. Also, CCS^dI^ alone was not able to activate the GAL4 transcription (no autoactivation). In this study, we screened 12.8 million prey clones for the interaction with CCS^dI^ and performed further analysis on 112 blue colonies grown on the plate in the presence of AbA. By sequencing of cDNA in those positive clones, the clones expressing non-authentic, artificial proteins such as frameshifted and intron-coded products were excluded: out of 15 clones coding proteins in frame, a hypothetical protein encoded in open reading frame 123 of chromosome 1, C1orf123, was found.

**Fig 1:**
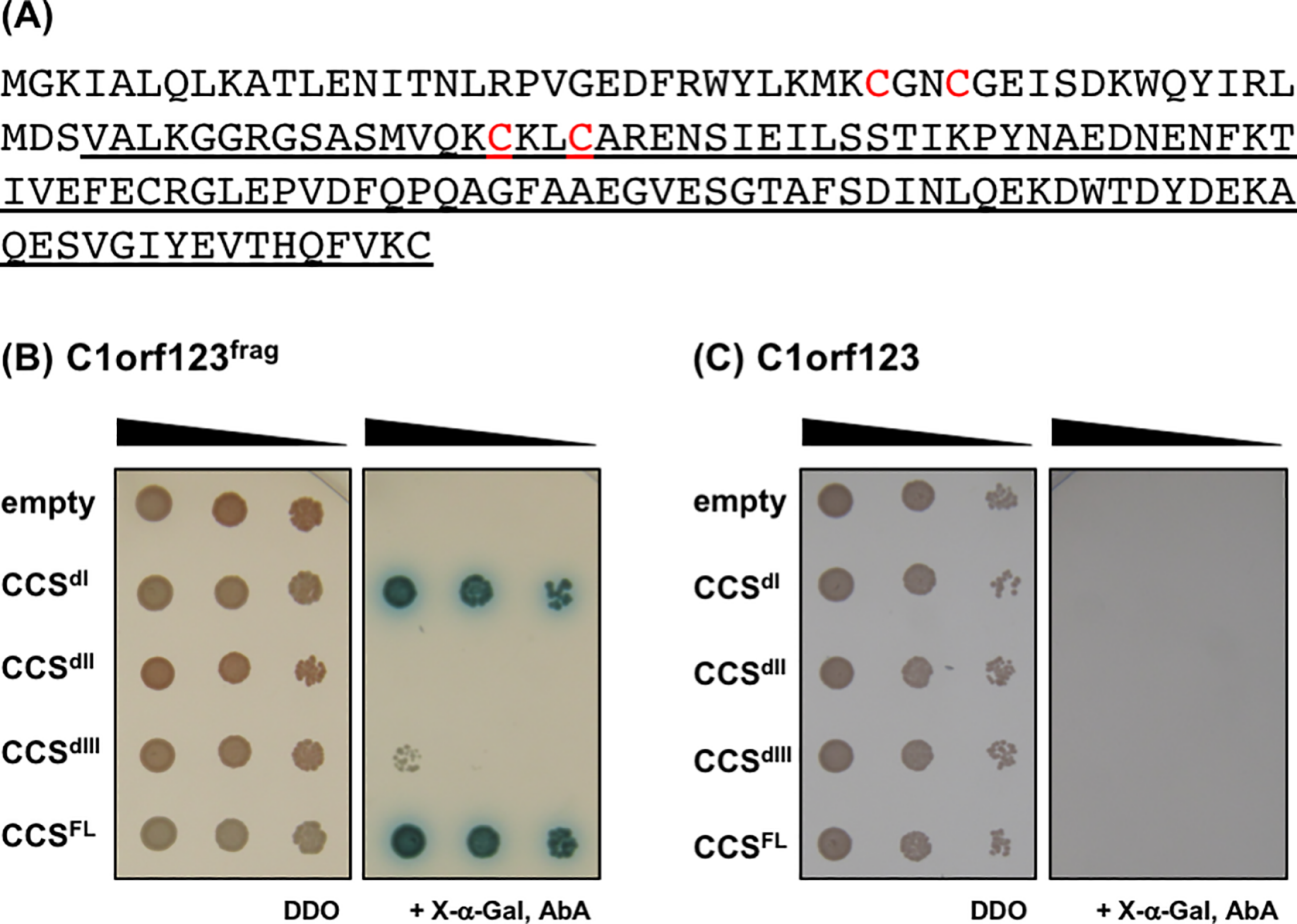
Yeast two-hybrid screening identifies C1orf123 as a protein interacting with CCS^dI^. **(A)** Amino acid sequence of C1orf123. An N-terminally truncated form of C1orf123 that was originally identified as a CCS^dI^-interacting partner (C1orf123^frag^) is underlined. Cys residues of the CxxC motifs in C1orf123 (Cys 33, 36, 67, 70) were colored red. **(B, C)** Interactions of CCS and its domains with (B) C1orf123^frag^ and (C) full-length C1orf123 were examined with a yeast two-hybrid system. Y2H-Gold yeast cells expressing CCS proteins were mated with Y187 yeast cells expressing C1orf123 proteins. Interaction of CCS proteins with C1orf123 allows the mated yeast cells to grow as blue-colored cells on a DDO medium in the presence of X-α-Gal and AbA (right panel in each figure). In the absence of X-α-Gal and AbA, the mated yeast cells can grow irrespective of the interaction between the proteins (left panel in each figure). The results were reproduced in three independent experiments.

While C1orf123 coded in the screened prey was not a full-length protein but was N-terminally truncated (C1orf123^frag^) (Fig 1A), the interaction was observed specifically with CCS^dI^ but not with CCS^dII^ and CCS^dIII^. When C1orf123^frag^ fused with GAL4 DNA BD was expressed with GAL4 AD (empty in Fig 1B), no blue-colored cells were grown in the presence of AbA, showing no autoactivation with C1orf123^frag^. C1orf123^frag^ also interacted with the full-length CCS protein (CCS^FL^) (Fig 1B), which was considered to occur through CCS^dI^. When the full-length C1orf123 protein was used instead of C1orf123^frag^, however, no interaction with all CCS proteins (CCS^dI^, CCS^dII^, CCS^dIII^, and CCS^FL^) was observed (Fig 1C). We thus supposed that the N-terminal truncation significantly impacted the structure of C1orf123 and allowed the interaction with CCS.

### Structural characterization of C1orf123 in solution

**Fig 2:**
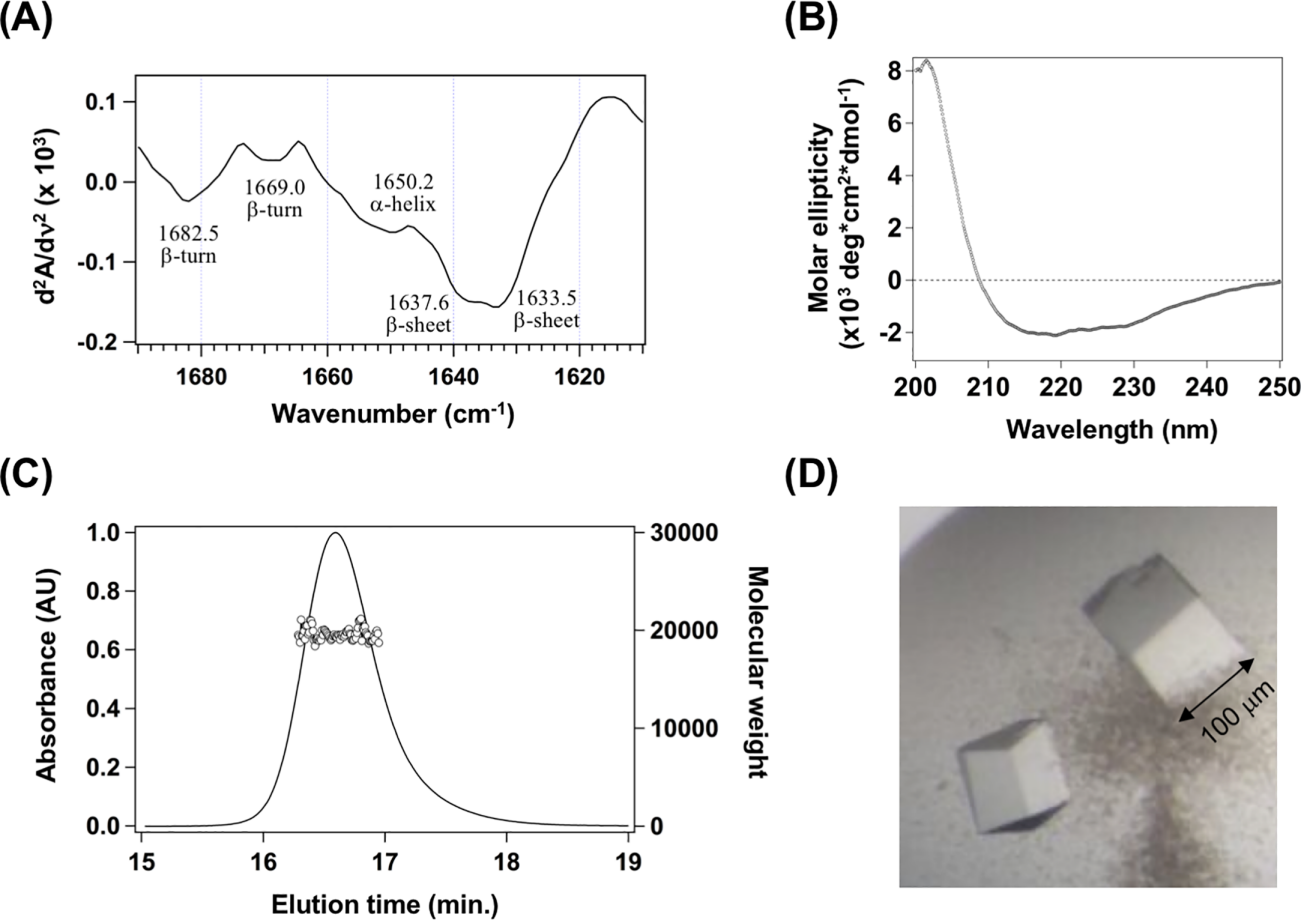
C1orf123 is a monomeric protein rich in β-sheet structures. **(A)** Second derivative FT-IR spectrum of C1orf123 in deuterated TN buffer. The assignment of absorption peaks to secondary structures was indicated in the figure. **(B)** CD spectrum of C1orf123 in a buffer containing 10 mM Na-Pi and 100 mM NaCl at pH 7.0. **(C)** Gel-filtration chromatogram of 2 g/L (*ca*. 100 μM) C1orf123 in TN buffer was obtained by monitoring the absorbance change at 280 nm (solid curve, left axis). The elution was analyzed by SEC-MALS, and the observed molecular weight was also shown (open circles, right axis). We confirmed that these results (A-C) were reproducible in at least two independent experiments. **(D)** Crystals of C1orf123 obtained by incubation at 4 ^o^C for a day.

We found that C1orf123^frag^ overexpressed in *E*. *coli* was insoluble, while full-length C1orf123 was obtained in a soluble fraction. CCS^dI^ would thus be presumed to recognize misfolded/denatured conformations of C1orf123. Based upon the primary sequence, C1orf123 belongs to Pfam family DUF866 [29] and shares 26% identity with a hypothetical protein, MAL13P1.257, from *Plasmodium falciparum*, which provides the only known three-dimensional structure in DUF866 family (PDB ID: 1ZSO) [30]. C1orf123 exhibits no significant sequence homology to any other proteins deposited in the protein data bank. To experimentally examine if our C1orf123 protein is structurally similar to MAL13P1.257, we examined the secondary structural properties of C1orf123 with Fourier-transformed infrared spectroscopy (FTIR). The second derivative FTIR spectrum of C1orf123 revealed absorption peaks at 1633.5 and 1637.6 cm^-1^, indicating the presence of β-sheet structures (Fig 2A). In addition, β-turns (1669.0 and 1682.5 cm^-1^) and α-helices (1650.2 cm^-1^) also appear to contribute to the structure of C1orf123 (Fig 2A). A structure rich in β-sheets was also supported by the circular dichroism (CD) spectrum of C1orf123, which was characterized by negative CD at 218 nm with intensely positive signal at 201 nm [31] (Fig 2B). These results hence confirmed our expectation that C1orf123 adopts a β-sheet-rich structure similar to that of MAL13P1.257.

As shown in Fig 2C, our purified C1orf123 eluted as a single peak in a gel-filtration chromatogram, and the MALS analysis on the eluted C1orf123 protein showed molecular mass of 20,000. Given that the calculated mass of C1orf123 (without an N-terminal His tag) is 18,203, C1orf123 is considered to be dominantly monomeric in solution. It has been reported that MAL13P1.257 is crystallized as a homodimer in an asymmetric unit and exists as a weak dimer in solution [30]. Taken together, C1orf123 and MAL13P1.257 contain similar contents of secondary structures but are characterized by distinct properties in their quaternary structures.

### Crystal structure analysis reveals that C1orf123 is a Zn^2+^-binding protein

**Fig 3:**
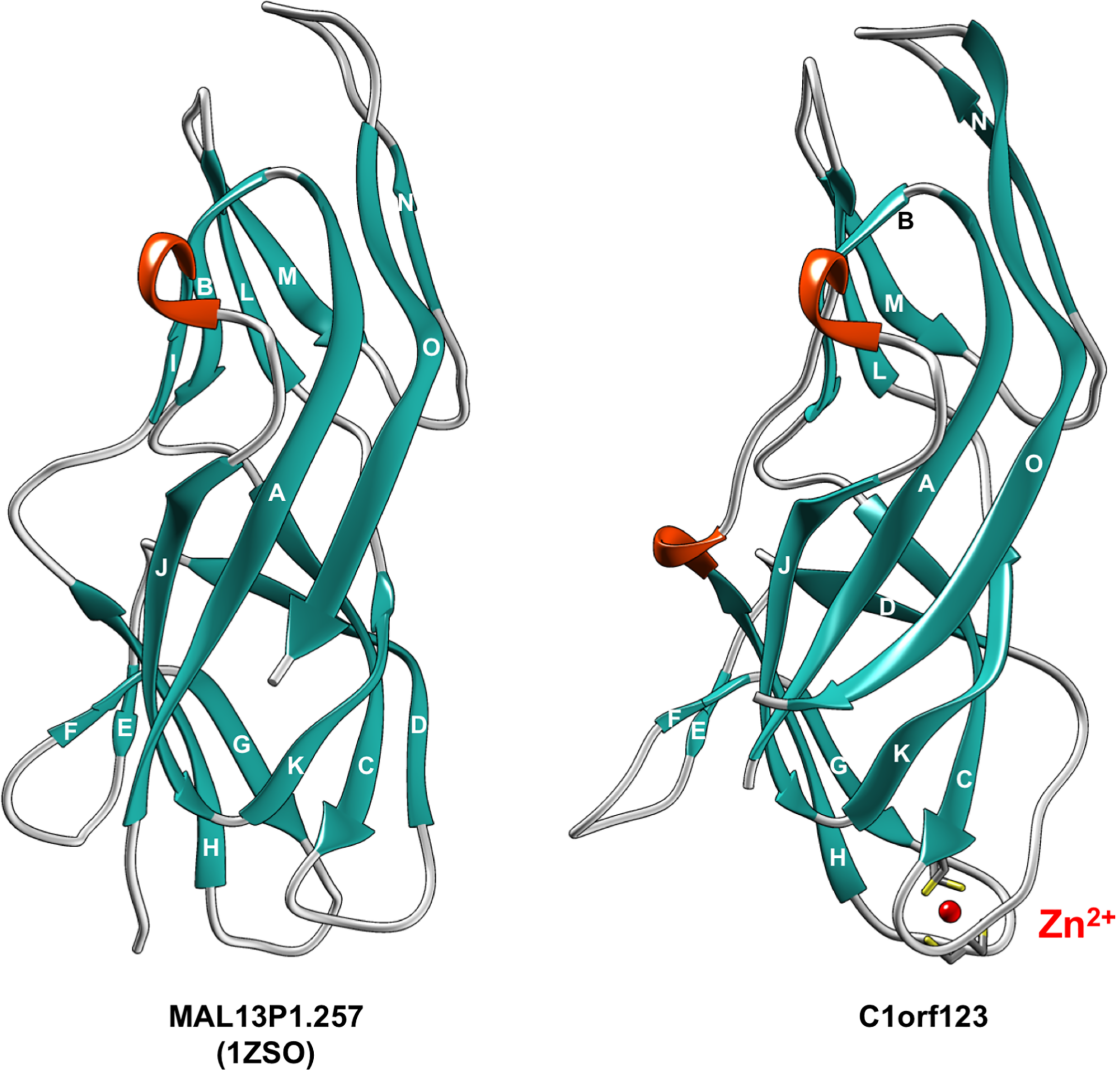
C1orf123 has a similar structure to that of MAL13P1.257. The structure of C1orf123 revealed in this study is shown as a ribbon model (right) and is compared with the structure of previously published MAL13P1.257 (PDB ID, 1ZSO: left). β-strands are colored in light sea green and designated in alphabetical order from A to O. Helical structures are colored orange. A Zn^2+^ ion (red ball) ligated with four Cys residues (stick model) is also shown for C1orf123.

The X-ray structural analysis of C1orf123 provides more insight into the structure of C1orf123. Purified C1orf123 gave cubic crystals within one day (also see the Materials and methods section) (Fig 2D). The phase was successfully determined due to the anomalous signal from a zinc ion in C1orf123, allowing us to solve the crystal structure at a resolution of 2.0 Å. Detailed procedures for structural determination were described in the Materials and methods section. As shown in Fig 3, the overall structure was found to be quite similar to that of MAL13P1.257 to the extent that the main chain Cα RMSD between those two proteins was 5.3 Å. Given that heterologous HMA domains form a metal-bridged symmetric dimer structure (*e*.*g*. [10]), C1orf123 was initially expected to have a structural region mimicking a βαββαβ folding pattern of a HMA domain for the interaction with CCS^dI^, which was, however, not the case. Also, CCS is known to interact with its substrate, SOD1, through its second domain (CCS^dII^), which has a quite similar folding pattern to that of SOD1 (*i*.*e*. a Greek key β-barrel structural motif) [15]. Although it remains obscure whether CCS^dI^ interacts with CCS^dII^/SOD1, we sought to find any structural similarities between C1orf123 and CCS^dII^/SOD1; but again, we failed to detect a Greek key motif in C1orf123. Instead, the overall folding pattern of C1orf123 and MAL13P1.257 is unique in that it is not observed in any other proteins registered in the protein data bank. The interaction of C1orf123 with CCS^dI^ is, therefore, considered to be distinct from those observed in a symmetric dimer formed by heterologous/homologous HMA domains and also in a homo/hetero complex of SOD1 and CCS.

In spite of such structural uniqueness, there are several differences in the structures between C1orf123 and MAL13P1.257. The most notable difference is that C1orf123 but not MAL13P1.257 binds a Zn^2+^ ion. An equimolar amount of a Zn^2+^ ion in C1orf123 was indeed confirmed with graphite furnace atomic absorption spectroscopy (841 μM Zn was detected in the solution containing 823 μM C1orf123). Our recombinant C1orf123 proteins were expressed in *E*. *coli* with normal Luria-Bertani broth and purified without no further addition of zinc salts. A Zn^2+^ ion is thus a strong candidate for the natively-bound metal ion in physiological C1orf123, but specification of the native metal ion awaits the metal analysis in C1orf123 isolated from human tissues. Indeed, we have supposed that the bound Zn^2+^ ion in C1orf123 could be replaced with a copper ion. The Zn^2+^-bound C1orf123 (20 μM) in TN buffer was incubated with equimolar amounts of either copper(II) sulfate (CuSO_4_) or copper(I) sulfite (Cu_2_SO_3_) for 30 min. at 37 ^o^C. Unbound metal ions were removed with centrifugal ultrafiltration, and the metal contents in the samples were then analyzed by graphite furnace atomic absorption spectroscopy. The Zn^2+^-bound C1orf123 contained almost stoichiometric amounts of Zn^2+^ ([C1orf123]/[Zn^2+^] = 0.88), which were retained even after addition of CuSO_4_ ([C1orf123]/[Zn^2+^] = 0.85). In contrast, amounts of Zn^2+^ ion in the C1orf123 sample were slightly decreased upon addition of Cu_2_SO_3_ ([C1orf123]/[Zn^2+^] = 0.67). In both cases (CuSO_4_ and Cu_2_SO_3_), significant amounts of copper were detected in the samples; more precisely, [C1orf123]/[copper ion] was found to be 0.96 and 0.78 for CuSO_4_ and Cu_2_SO_3_, respectively. Therefore, the added copper ions would be adventitiously associated with the protein, but the reduction of the zinc content in the C1orf123 sample upon addition of Cu_2_SO_3_ might indicate the replacement of the bound Zn^2+^ ion with Cu^+^ ion.

**Fig 4:**
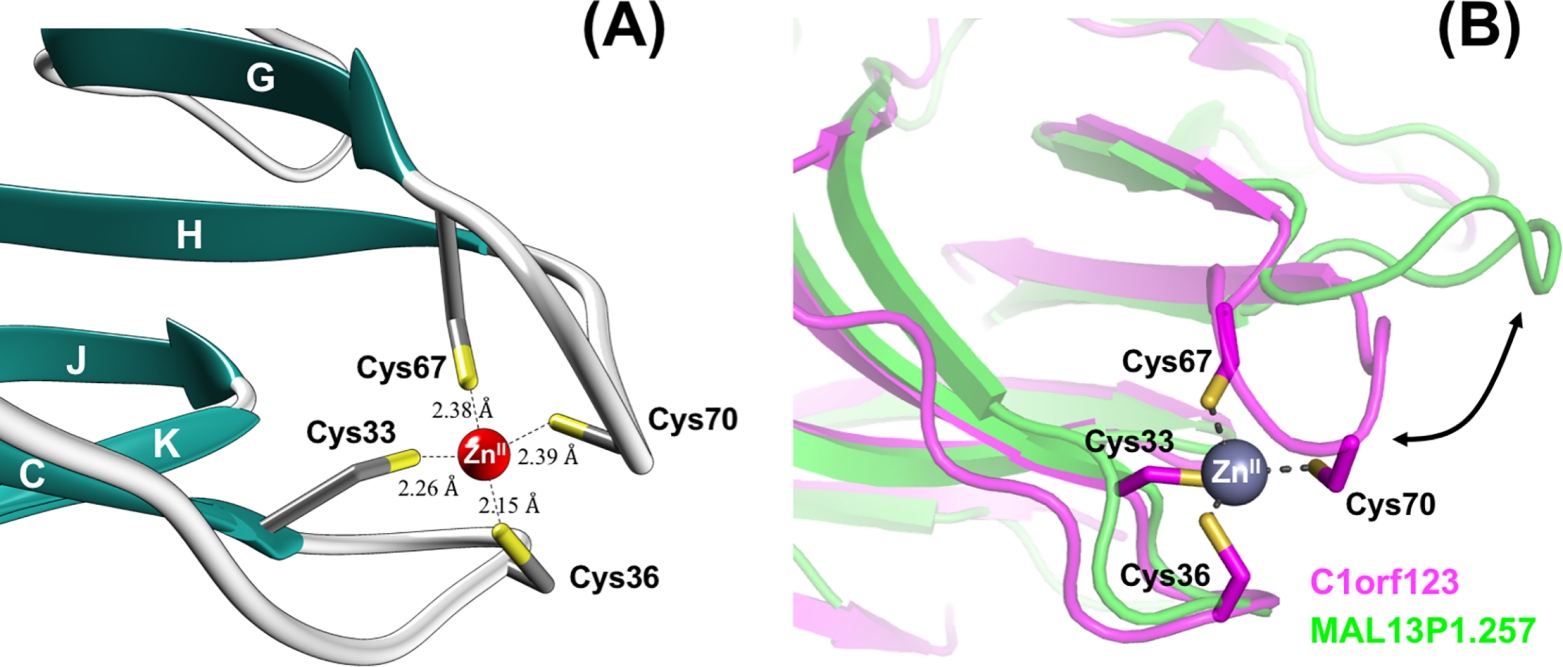
Close-up view of the Zn^2+^ binding site in C1orf123. **(A)** Tetrahedral coordination of a Zn^2+^ ion (a red ball) with four Cys residues (stick model) is shown with four Zn^2+^-Cys distances. **(B)** Structures of C1orf123 (magenta) and MAL13P1.257 (green) are aligned and superimposed. A Zn^2+^ ion and four Cys ligands are shown as a gray ball and sticks, respectively. The loop displaced between C1orf123 and MAL13P1.257 is indicated by an arrow.

In our crystal structure (Fig 4A), C1orf123 appears to sandwich the Zn^2+^ ion with two loops, each of which contains a CxxC motif. More precisely, the Zn^2+^ ion is ligated in tetrahedral coordination by four sulfur atoms in Cys33, Cys36, Cys67, and Cys70, with typical Zn^2+^-S bond lengths ranging from 2.15 to 2.39 Å (Fig 4A) [32]. In addition, a bond angle (∠S-Zn^2+^-S) ranges from 100^o^ to 117^o^, indicating the symmetrical tetrahedral coordination of a Zn^2+^ ion. MAL13P1.257 has no CxxC motif and is hence unlikely to bind metal ions at the site corresponding to the CxxC motifs in C1orf123. As shown in Fig 4B, the loop with Cys33 and Cys36 in C1orf123 (colored magenta) is located at similar position to that of MAL13P1.257 (colored light green), but the other loop with Cys67 and Cys70 in C1orf123 moves toward the Zn^2+^ ion so as to wrap it around. Regarding the Zn^2+^ binding in C1orf123, it should be also noted that C1orf123 was crystallized as a Zn^2+^-bound form in the crystallization solution at low pH (pH 4.5). Protonation of thiolate groups in Cys ligands generally facilitates the dissociation of the bound metal ions from proteins, but as of now, forty seven entries have been registered in the protein data bank (www.wwpdb.org) as protein crystal structures retaining Zn^2+^ ions through Cys ligands at pH lower than 4.5. While we could not pinpoint the exact reason for the stable Zn^2+^-Cys interaction in C1orf123 at acidic pH, four Cys ligands comprising of the Zn^2+^ coordination sphere may be somehow protected from the protonation.

**Fig 5:**
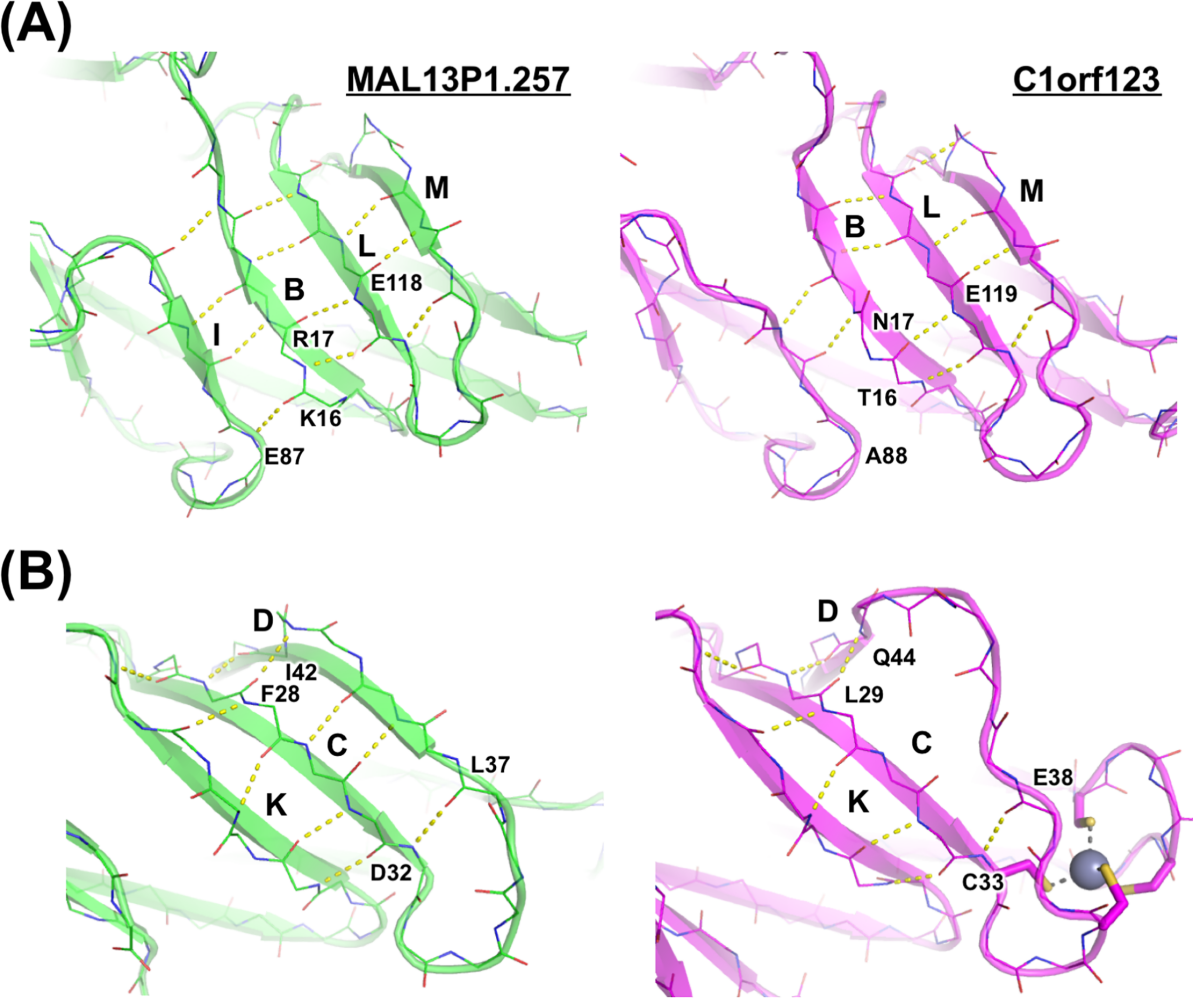
Close-up views describing structural differences in β-sheets between C1orf123 and MAL13P1.257. β-sheets comprised of **(A)** strands I/B/M/L, and **(B)** strands D/C/K are compared between MAL13P1.257 (green) and C1orf123 (magenta). Hydrogen bonds between carbonyl and amide groups of main chains are shown as broken lines colored yellow.

We also noticed differences in the secondary structures between C1orf123 and MAL13P1.257. Based upon secondary structures assigned by using a STRIDE software [33], fifteen β-strands (from A to O) and one 3_10_ helix can be identified in MAL13P1.257 (Fig 3), while C1orf123 lacks strand I and has an additional 3_10_ helical structure (Ser80 –Thr82). Both of those proteins form four antiparallel β-sheets, each of which is composed of the following β-strands: G/H/J/A/O, D/C/K, A/O/N, and I/B/L/M (Fig 3). Among them, strand I constitutes an antiparallel β-sheet in MAL13P1.257 but not in C1orf123 (Fig 3). Actually, the region corresponding to strand I is not assigned as a β-strand in C1orf123, since hydrogen bonds between strands I and B at their edges are too distant to be formed (Fig 5A, right). A main reason for this is flipping of the carbonyl group of Asn17 in C1orf123, which precludes the hydrogen bonding interaction with the amide group of Glu119 in strand L (Fig 5A, right). Instead, the amide group of Glu119 forms a hydrogen bond with the carbonyl group of Thr16, which is hence no longer available for the hydrogen bonding interaction with the amide group of Ala88 (Fig 5A, right). In the corresponding positions of MAL13P1.257, in contrast, the carbonyl group of Arg17 is in-register and forms the hydrogen bond with the amide group of Glu118 in strand L (Fig 5A, left). The carbonyl group of Lys16 can thus form the hydrogen bond with the amide group of Glu87, extending the β-sheet structure to strand I in MAL13P1.257 (Fig 5A, left).

In addition, strand D in C1orf123 is significantly shorter than that in MAL13P1.257 (Fig 3). Comparison of the D/C/K β-sheet region in C1orf123 and MAL13P1.257 shows that there is one extra amino acid residue between Glu38 and Gln44 in C1orf123 (Fig 5B). Similar to a hydrogen bond between the carbonyl oxygen of Leu37 and amide nitrogen of Asp32 in MAL13P1.257 (Fig 5B, left), Cys33 forms a hydrogen bond with Glu38 in C1orf123 (Fig 5B, right). Also, MAL13P1.257 forms a hydrogen bond between Phe28 and Ile42, which corresponds to a hydrogen bond between Leu29 and Gln44 in C1orf123. Due to an insertion of one amino acid residue in C1orf123 as compared with MAL13P1.257, however, amino acid residues at position 39–43 in C1orf123 cannot interact with strand C (Fig 5B, right). Such structural differences results in the short strand D in C1orf123.

Taken together, structures of C1orf123 and MAL13P1.257 are quite similar except that only C1orf123 binds a Zn^2+^ ion. We therefore supposed limited effects of Zn^2+^ ion on the structure of C1orf123; however, incubation of C1orf123 with a strong chelator for divalent heavy metal ions, ethylenediaminetetraacetic acid (EDTA) and *N*,*N*,*N'*,*N'*-Tetrakis(2-pyridylmethyl)ethylenediamine (TPEN), resulted in the precipitation of C1orf123 (S1 Fig), while the atomic absorption analysis showed that Zn^2+^ ion bound in C1orf123 remained in the supernatant. These results suggest the insolubilization of C1orf123 upon dissociation of the bound Zn^2+^ ion, which is further supported by the fact that C1orf123 becomes insoluble by substituting Ala for all four Cys ligands (Cys33, 36, 67, and 70) upon its overexpression in *E*. *coli*. It is thus expected that the binding of a Zn^2+^ ion through the CxxC motifs play essential roles in the structure/function of C1orf123; indeed, the dual CxxC motifs in C1orf123 are completely conserved among various species such as fungi, plants, worms, flies, and vertebrates (S2 Fig).

### Specific interaction between CCS^dI^ and C1orf123

**Fig 6:**
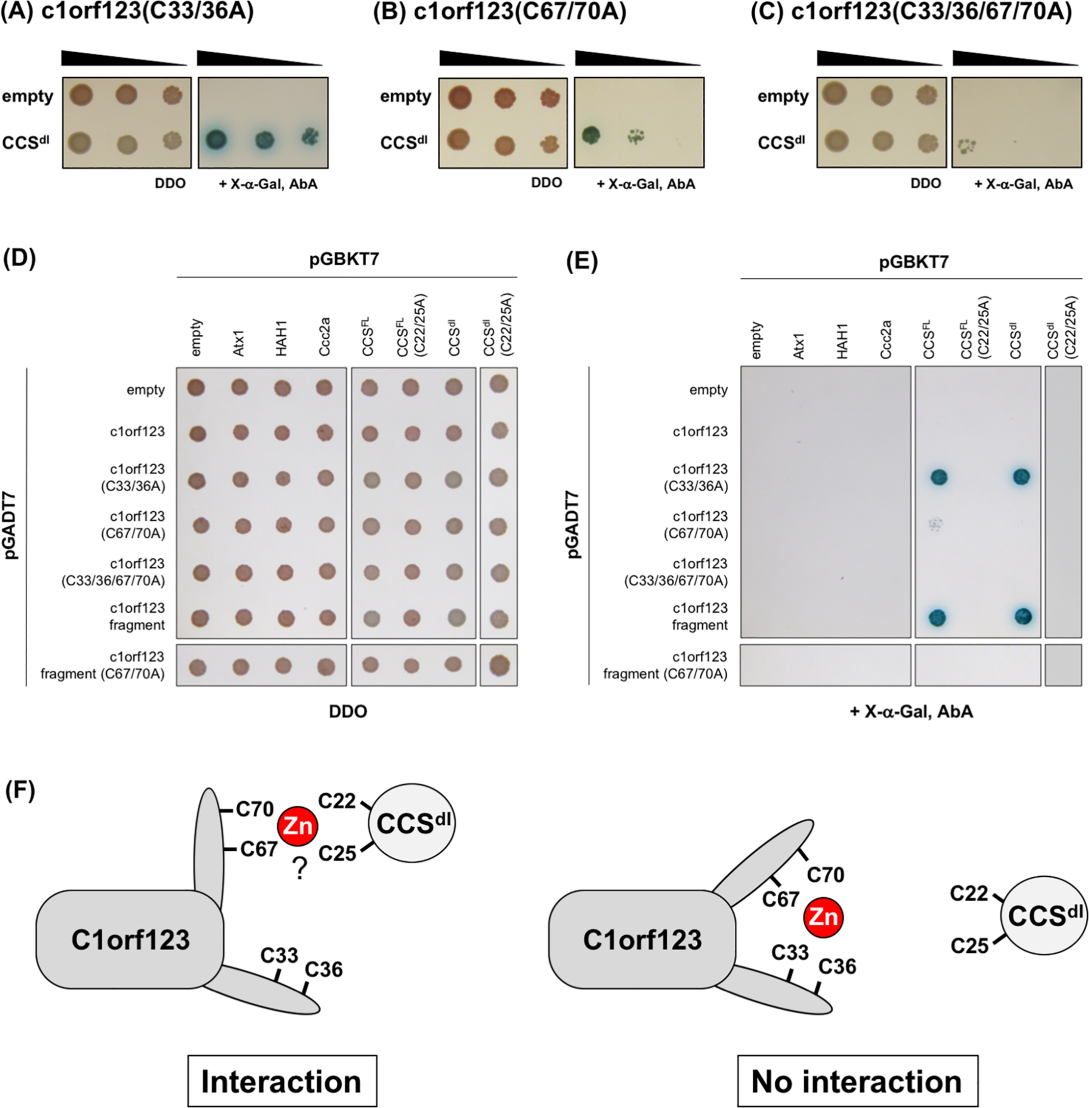
Specific interaction of C1orf123 with CCS^dI^ through the CxxC motifs. Interaction of CCS^dI^ with **(A)** C1orf123(C33/36A), **(B)** C1orf123(C67/70A), and **(C)** C1orf123(C33/36/67/70A) was examined by yeast two-hybrid system using Y2H-Gold and Y187 yeast cells as described in Fig 1. Mated cells were confirmed by incubation on DDO plates (left panel), while the interaction between the proteins was evaluated by spotting the mated cells on DDO plates in the presence of X-α-Gal and AbA (right panel). **(D, E)** C1orf123 and its indicated mutant forms were cloned in pGADT7 and expressed in Y187 yeast cells, which were further mated with Y2H-Gold expressing CCS, CCS^dI^, and proteins containing a HMA domain (Atx1, HAH1, and Ccc2a) cloned in pGBKT7. The mated cells were confirmed on a DDO plate (D), and the interactions between the proteins expressed were examined on a DDO plate with X-α-Gal and AbA (E). The results were reproduced in three independent experiments, but in spite of the same experimental procedure between panels B and E, yeast cells expressing CCS^dI^ and C1orf123(C67/70A) appeared to grow (B) or not to grow (E) on the DDO plate with X-α-Gal and AbA. We suppose that this inconsistency is due to the longer incubation time in panels A/B/C than that in panel E. Indeed, there were colonies in very small size, albeit almost invisible in panel (E), in the CCS^dI^/C1orf123(C67/70A) yeast. **(F)** Schematic representation of the CxxC-dependent interaction between C1orf123 and CCS^dI^.

Given that binding of a Zn^2+^ ion is required for maintaining the folded structure of C1orf123, truncation of the N-terminal region covering one of the two CxxC motifs (C^33^xxC^36^) is expected to preclude Zn^2+^ binding and result in denaturation of C1orf123^frag^ (Fig 1A). The truncated but not full-length C1orf123 was able to interact with CCS^dI^ (Fig 1B and 1C); therefore, the zinc binding in C1orf123 is expected to inhibit the interaction with CCS^dI^. We thus introduced Cys-to-Ala mutations in the CxxC motifs of C1orf123 and examined the interaction of those mutated C1orf123 proteins with CCS^dI^ in yeast two-hybrid experiments. As shown in Fig 6A, the Cys-to-Ala mutations at C^33^xxC^36^ in full-length C1orf123 restored the interaction with CCS^dI^. Also, C1orf123 was able to interact with CCS^dI^ by introducing the mutations at C^67^xxC^70^ (C67/70A), albeit to a lesser extent (Fig 6B). These results support our expectation that C1orf123 will interact with CCS^dI^ upon dissociation of the bound Zn^2+^ ion. When all of those four Cys residues (Cys33, 36, 67, and 70) were mutated to Ala, C1orf123 presumably binds no metal ions and was also no longer able to interact with CCS^dI^ (Fig 6C). This is also confirmed by no interaction of CCS^dI^ with C67/70A-mutant C1orf123^frag^ (Fig 6D and 6E). These data hence indicate that C1orf123 can interact with CCS^dI^ when either one of the two CxxC motifs (C^67^xxC^70^ in particular) is available (Fig 6F).

When Ala were substituted for Cys residues in the CxxC motif of CCS^FL^/CCS^dI^, the interactions of such mutant CCS proteins with C33/36A-mutant C1orf123 and C1orf123^frag^ were not observed (Fig 6D and 6E). We thus suggest a CxxC-dependent complexation of CCS^dI^ with C1orf123, in which a metal ion is possibly ligated with the CxxC motifs from CCS^dI^ and C1orf123 (Fig 6F). Given that the crystal structure reveals the binding of Zn^2+^ ion in C1orf123 and also that CCS^dI^ has ability to bind a Zn^2+^ ion (*vide infra*), we expect the CxxC-dependent complexation between C1orf123 and CCS in a Zn^2+^-mediated fashion; however, there is a caveat that the metal ion involved in the complexation is yet to be positively identified. It is also notable that C33/36A-mutant C1orf123 did not interact with other proteins composed of a HMA domain such as Atx1, HAH1, and Ccc2a (Fig 6D and 6E). While these proteins have a CxxC motif and a similar fold to that of CCS^dI^, no interactions with the mutant C1orf123 support high specificity in the recognition between CCS^dI^ and C1orf123.

### Possible roles of C1orf123 and its relation to copper chaperones

**Fig 7:**
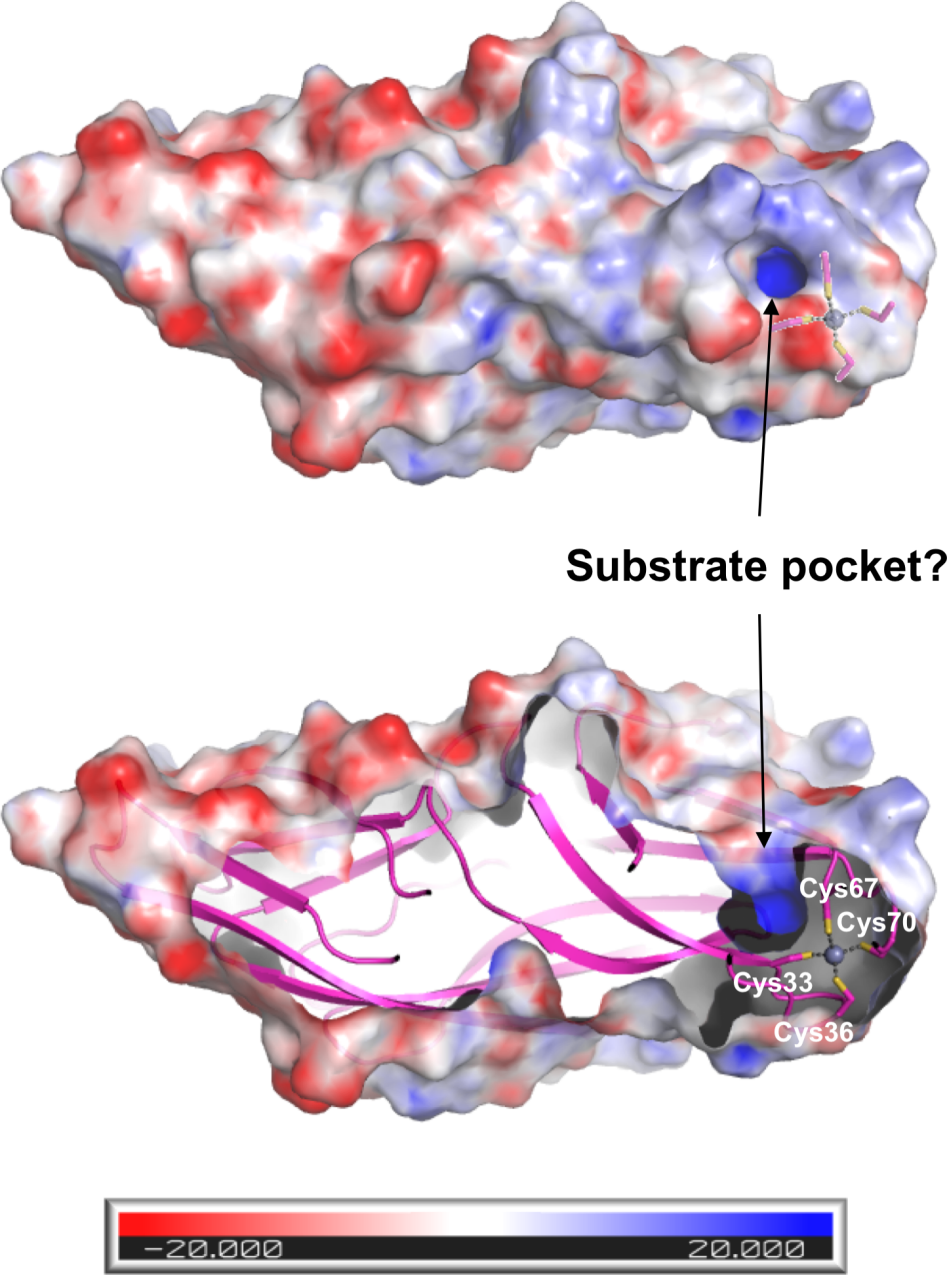
Structural features of C1orf123 that might be related to its function. In electrostatic potential of the surface of C1orf123, the positive and negative charges are colored with blue and red, respectively, according to the scale shown below. The unit of the scale is *kT*/*e*, where *k*, T, and *e* are Boltzmann’s constant, temperature, and the electronic charge, respectively (top). The cross section of the protein was also shown to clarify the pocket located adjacent to the Zn^2+^ ion, which is indicated with four Cys ligands in a ball and stick model (bottom). The pocket volume was estimated to be 214 Å^3^ using a POCASA program with probe radius of 2.0 Å.

While functions of C1orf123 remain completely unknown, several features on the structure of C1orf123 should be noted. C1orf123 is like a rugby ball in its shape and has characteristic charge distribution on its surface (Fig 7); the zinc-containing side of the protein is positively charged, while the other side concentrates negative charges. Such charge distribution might have roles in specific interactions of C1orf123 with biomolecules for certain reactions. As shown in Fig 7, we also found the positively charged pocket adjacent to the bound Zn^2+^ ion. The volume of this pocket is calculated as 214 Å^3^ using a POCASA program with probe radius of 2.0 Å [34], which can accommodate substrates of significant size. In most zinc-containing enzymes, however, the catalytic Zn^2+^ is ligated by three Cys/His residues and the substrate/water molecule [32]; therefore, a tetrahedral coordination of Zn^2+^ with four Cys residues in C1orf123 would not fit with a role of the Zn^2+^ ion as a catalytic site. Alternatively, as seen in C4-type zinc fingers [32], physiological functions of C1orf123 may require the binding of a Zn^2+^ ion, which has a structural role in maintaining the functionally competent conformation (S1 Fig). We also found significant fluctuations specifically in the loop (Arg47 –Ser62) of C1orf123, which might have functional roles in C1orf123 (S3 Fig).

As mentioned above, a Zn^2+^ ion may not be a “natively-bound” metal ion for C1orf123 under physiological conditions, and also, CCS is well established as a copper-binding protein (namely, a copper chaperone protein). Despite this, copper chaperones comprised of HMA domains have been shown to tightly bind a Zn^2+^ ion as well as a Cu^+^ ion and form a Zn^2+^-mediated heterocomplex with the HMA domain of their partner proteins (*e*.*g*. [35]). For example, a human copper chaperone HAH1 is a copper-binding HMA domain protein and forms a tight Zn^2+^-mediated complex with the HMA domain of its physiological partner ATP7a/b [36]. Similar to those copper chaperones binding a Zn^2+^ ion, recombinant CCS^dI^ was found to retain significant amounts of Zn^2+^ ion (12 μM Zn^2+^ in 20 μM CCS^dI^) when incubated with equimolar amounts of Zn^2+^ ion followed by vigorous buffer exchange with centrifugal ultrafiltration. We thus expected the transfer of a Zn^2+^ ion from CCS^dI^ to apo-C1orf123 through their specific interaction, but its experimental test was difficult due to the highly insoluble nature of apo-C1orf123 (S1 Fig). As summarized in S2 Fig, furthermore, fruit fly (*D*. *melanogaster*) and mosquito (*A*. *gambiae*) have C1orf123, but their CCS proteins are known to lack the CxxC motif in CCS^dI^ [19]. A nematode (*C*. *elegans*) also has C1orf123 (S2 Fig) but no CCS, suggesting that Zn^2+^-bound CCS^dI^ is not essential in the supply of a Zn^2+^ ion to C1orf123. A role of CCS in the maturation of C1orf123 as a zinc chaperone is attractive but currently speculative and thus needs to be tested in a future.

According to the human proteome map [37], expression of C1orf123 is confirmed in ovary, testis, pancreas, urinary bladder prostate, and CD8+ T cells. In contrast, expression levels of C1orf123 are low in tissues such as brain, spinal cord, heart, liver, lung, and kidney. Actually, we attempted to detect C1orf123 in human embryonic kidney cells 293 (HEK293) and mouse neuroblastoma cells (*Neuro*-2a) but failed, which probably reflects low expression levels of C1orf123 in kidney and brain. C1orf123 is thus expected to perform some tissue-specific functions, but as of now, no definitive conclusions can be drawn on the function of C1orf123. We are now exploring the function of this newly identified zinc-binding protein, C1orf123.

## Supporting information

S1 Fig Precipitation of C1orf123 upon treatment with Zn2+ chelator. C1orf123 (20 μM) in TN buffer was incubated in the absence (-) and presence of Zn^2+^ chelators, TPEN (1 mM) and EDTA (5 mM), at 37 ^o^C for 20 hours. The samples were then centrifuged at 20,000 x *g* for 10 min to fractionate into soluble supernatant (s) and insoluble pellet (i) and then analyzed with SDS-PAGE.
(TIFF)

S2 Fig Multiple alignment for amino acid sequences of C1orf123 and its representative homologs from indicated species. Alignment was performed using ClustalW. Human C1orf123 is colored red, and the conserved two CxxC motifs were highlighted in yellow. Together with information on the amino acid sequence, the species indicated in the figure are as follows:
Human: *Homo sapiens* (NP_060357.1)
Chimpanzee: *Pan troglodytes* (XP_009439533.2)
Rhesus macaque: *Macaca mulatta* (NP_001253531.1)
Bovine: *Bos Taurus* (NP_001033219.1)
Dog: *Canis lupus familiaris* () (XP_536703.3)
Rat: *Rattus norvegicus* (NP_001029304.1)
Mouse: *Mus musculus* (NP_001334089.1)
Chicken: *Gallus gallus* (XP_015146700.1)
Zebrafish: *Danio rerio* (NP_001122157.1)
Fruit fly: *Drosophila melanogaster* (NP_001286382.1)
Mosquito: *Anopheles gambiae str*. *PEST* (XP_318370.4)
*C*. *elegans*: *Caenorhabditis elegans* (NP_505521.2)
Mouse-ear cress: *Arabidopsis thaliana* (NP_567911.1)
Rice: *Oryza sativa subsp*. *Japonica* (XP_015650849.1)
*M*. *oryzae*: *Magnaporthe oryzae 70–15* (XP_003709416.1)
Red bread mold: *Neurospora crassa OR74A* (XP_963434.1)
Baker’s yeast: *Saccharomyces cerevisiae* (NP_010014.1)
Fission yeast: *Schizosaccharomyces pombe* (NP_001018829.2)
*K*. *lactis*: *Kluyveromyces lactis* (XP_455542.1)
*E*. *gossypii*: *Eremothecium gossypii ATCC 10895* (NP_986429.
(TIFF)

S3 Fig Fluctuations of overall structure of C1orf123. The X-ray structure with rainbow colors according to the B-factor values for Cα atoms. The loop (Arg47 –Ser62) with yellow and red colors have higher B-factor values (~50 Å^2^) than the other region.
(TIFF)
