# Supplementary figures and images for: Identification of a novel zinc-binding protein, C1orf123, as an interactor with a heavy metal-associated domain
Source: PLoS One. 2018 Sep 27;13(9):e0204355. doi: 10.1371/journal.pone.0204355 (PMC6160046; doi:10.1371/journal.pone.0204355)

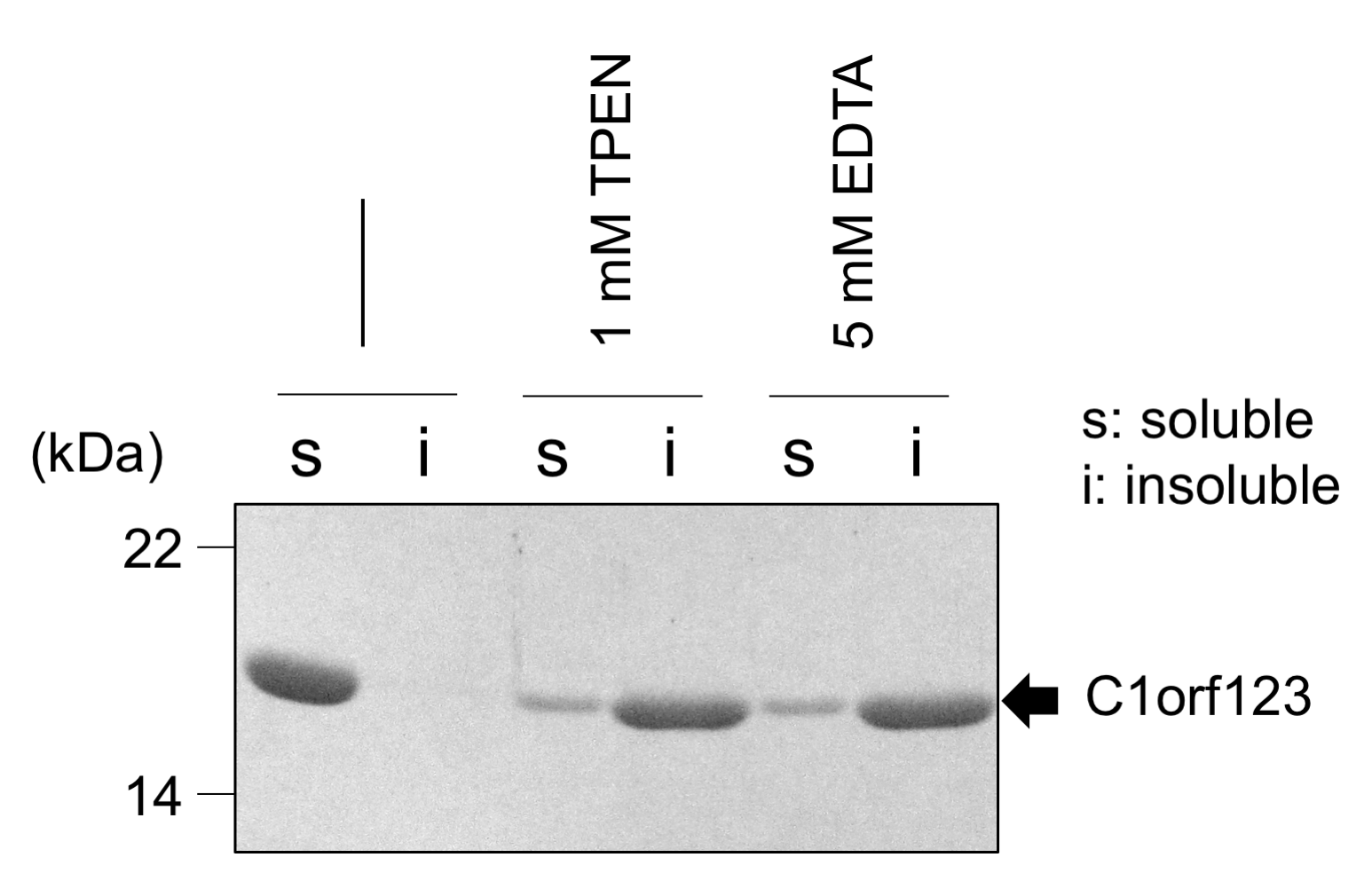

Supplement: S1 Fig — C1orf123 (20 μM) in TN buffer was incubated in the absence (-) and presence of Zn2+ chelators, TPEN (1 mM) and EDTA (5 mM), at 37 oC for 20 hours. The samples were then centrifuged at 20,000 x g for 10 min to fractionate into soluble supernatant (s) and insoluble pellet (i) and then analyzed with SDS-PAGE. (TIFF) [file pone.0204355.s001.tiff]

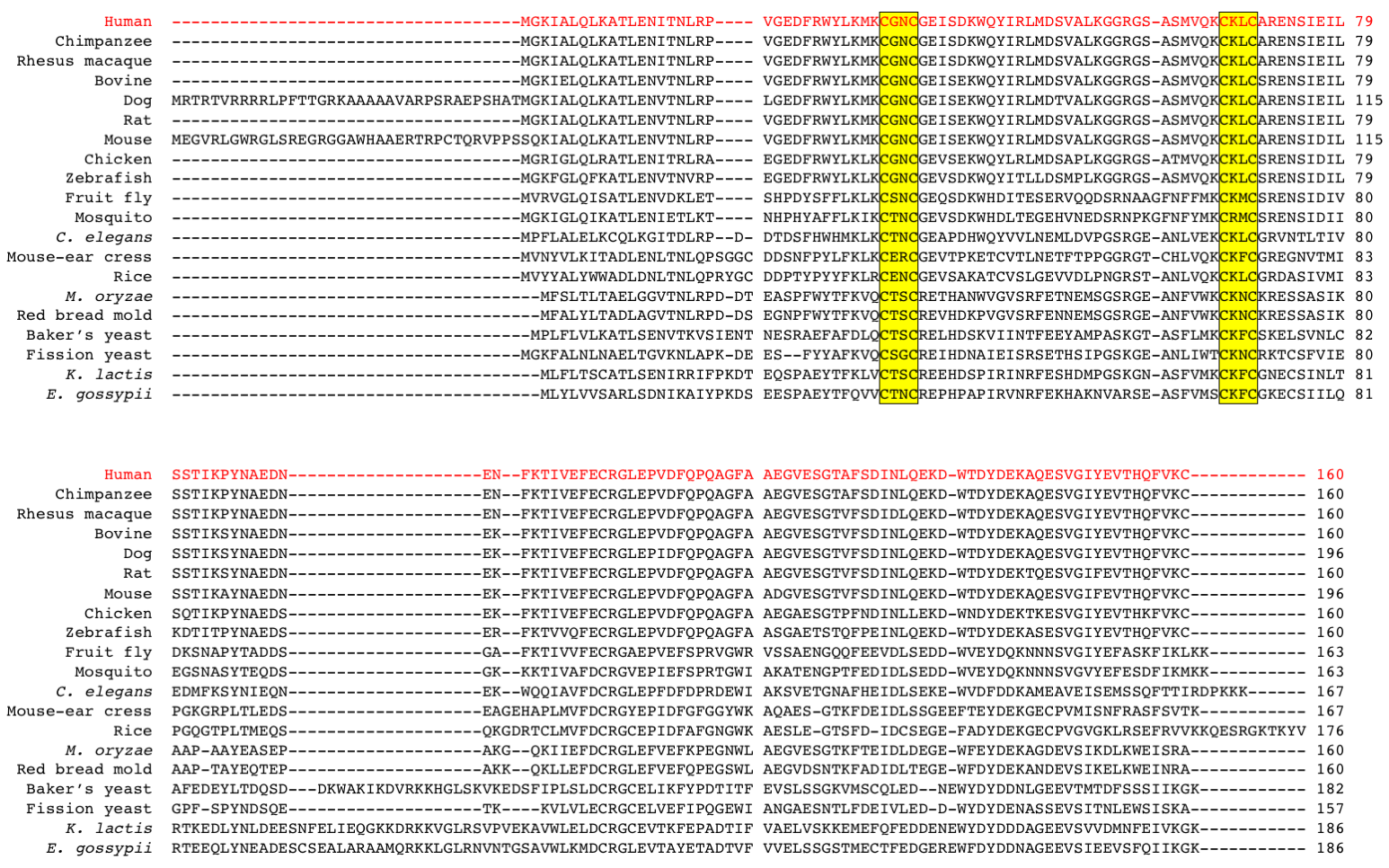

Supplement: S2 Fig — Alignment was performed using ClustalW. Human C1orf123 is colored red, and the conserved two CxxC motifs were highlighted in yellow. Together with information on the amino acid sequence, the species indicated in the figure are as follows: Human: Homo sapiens (NP_060357.1) Chimpanzee: Pan troglodytes (XP_009439533.2) Rhesus macaque: Macaca mulatta (NP_001253531.1) Bovine: Bos Taurus (NP_001033219.1) Dog: Canis lupus familiaris () (XP_536703.3) Rat: Rattus norvegicus (NP_001029304.1) Mouse: Mus musculus (NP_001334089.1) Chicken: Gallus gallus (XP_015146700.1) Zebrafish: Danio rerio (NP_001122157.1) Fruit fly: Drosophila melanogaster (NP_001286382.1) Mosquito: Anopheles gambiae str. PEST (XP_318370.4) C. elegans: Caenorhabditis elegans (NP_505521.2) Mouse-ear cress: Arabidopsis thaliana (NP_567911.1) Rice: Oryza sativa subsp. Japonica (XP_015650849.1) M. oryzae: Magnaporthe oryzae 70–15 (XP_003709416.1) Red bread mold: Neurospora crassa OR74A (XP_963434.1) Baker’s yeast: Saccharomyces cerevisiae (NP_010014.1) Fission yeast: Schizosaccharomyces pombe (NP_001018829.2) K. lactis: Kluyveromyces lactis (XP_455542.1) E. gossypii: Eremothecium gossypii ATCC 10895 (NP_986429. (TIFF) [file pone.0204355.s002.tiff]

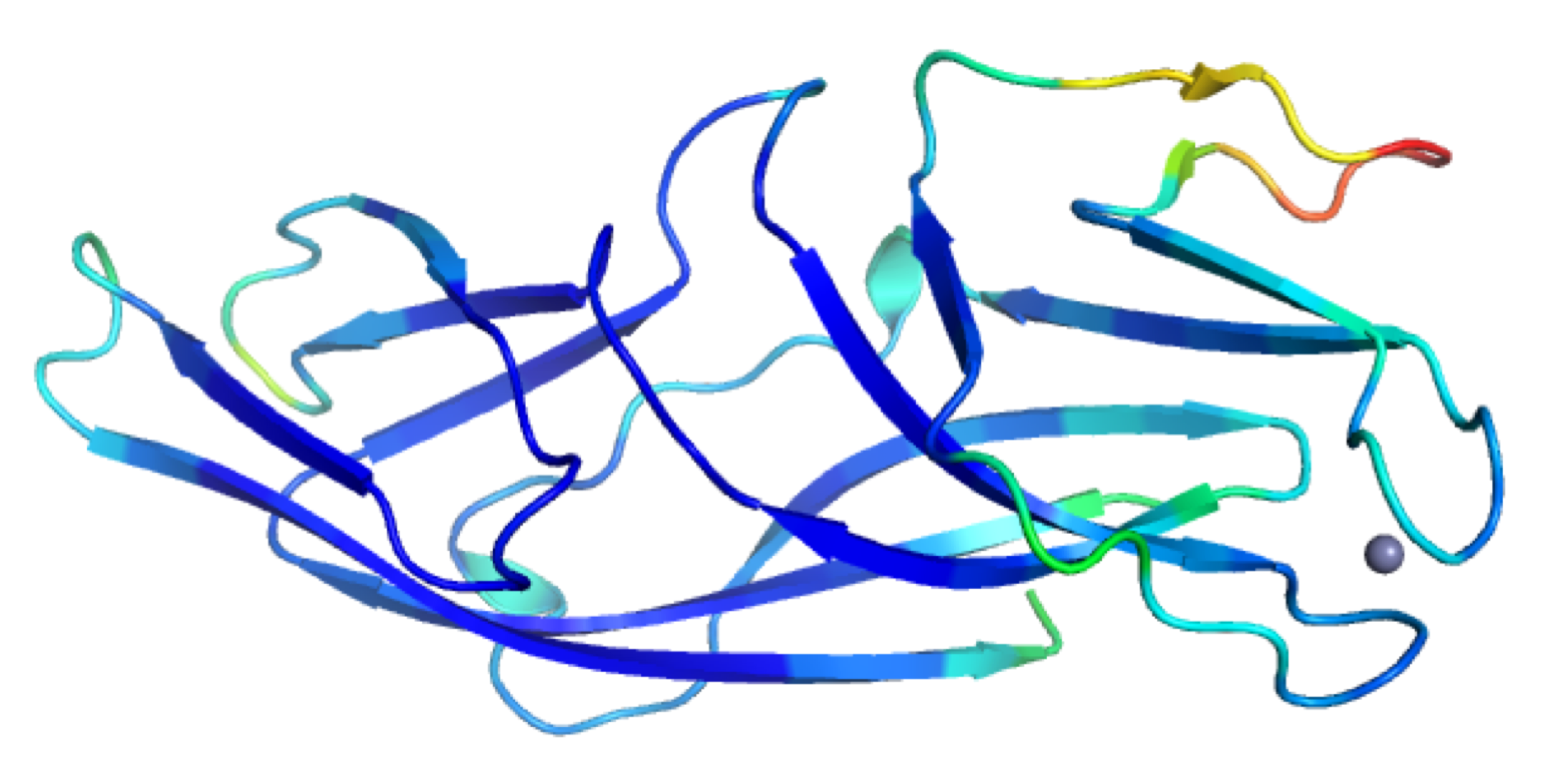

Supplement: S3 Fig — The X-ray structure with rainbow colors according to the B-factor values for Cα atoms. The loop (Arg47 –Ser62) with yellow and red colors have higher B-factor values (~50 Å2) than the other region. (TIFF) [file pone.0204355.s003.tiff]
